# Development of a One-Step Immunocapture Real-Time RT-PCR Assay for Detection of *Tobacco Mosaic Virus* in Soil

**DOI:** 10.3390/s121216685

**Published:** 2012-12-04

**Authors:** Jin-Guang Yang, Feng-Long Wang, De-Xin Chen, Li-Li Shen, Yu-Mei Qian, Zhi-Yong Liang, Wen-Chang Zhou, Tai-He Yan

**Affiliations:** 1 Open Project Program of Key Laboratory of Tobacco Pest Monitoring Controlling & Integrated Management, Tobacco Research Institute of CAAS, Qingdao 266101, China; E-Mails: jinguangyang@126.com (J.-G.Y.); cdxycs@gmail.com (D.-X.C.); sdrzsll@tom.com (L.-L.S.); qymycs@sohu.com (Y.-M.Q.); 2 Shandong Weifang Tobacco Co., Ltd., Weifang 250100, China; E-Mails: clyc2009@163.com (Z.-Y.L.); clycsc@126.com (W.-C.Z.); ythhe003@126.com (T.-H.Y.)

**Keywords:** *Tobacco mosaic virus*, immunocapture qRT-PCR, detection, soil

## Abstract

*Tobacco mosaic virus* (TMV) causes significant losses in many economically important crops. Contaminated soils may play roles as reservoirs and sources of transmission for TMV. In this study we report the development of an immunocapture real-time RT-PCR (IC-real-time RT-PCR) assay for direct detection of TMV in soils without RNA isolation. A series of TMV infected leaf sap dilutions of 1:10^1^, 1:10^2^, 1:10^3^, 1:10^4^, 1:10^5^ and 1:10^6^ (w/v, g/mL) were added to one gram of soil. The reactivity of DAS-ELISA and conventional RT-PCR was in the range of 1:10^2^ and 1:10^3^ dilution in TMV-infested soils, respectively. Meanwhile, the detection limit of IC-real-time RT-PCR sensitivity was up to 1:10^6^ dilution. However, in plant sap infected by TMV, both IC-real-time RT-PCR and real-time RT-PCR were up to 1:10^6^ dilution, DAS-ELISA could detect at least 1:10^3^ dilution. IC-real-time RT-PCR method can use either plant sample extracts or cultivated soils, and show higher sensitivity than RT-PCR and DAS-ELISA for detection of TMV in soils. Therefore, the proposed IC-real-time RT-PCR assay provides an alternative for quick and very sensitive detection of TMV in soils, with the advantage of not requiring a concentration or RNA purification steps while still allowing detection of TMV for disease control.

## 1. Introduction

*Tobacco mosaic virus* (TMV) is one of the most devastating viruses that infects many crops worldwide, including vegetables, ornamentals, legumes, and other important crops, especially tobacco and other members of the family *Solanaceae*. Since its description in the late 19th century, diseases caused by TMV continue to cause heavy agricultural losses worldwide [1]. TMV is a positive, single stranded RNA virus, with a total genome of approximately 6,500 bp in length which is composed of three open reading frames (ORFs) [2]. As one of the most stable viruses, it has a very wide host range. The persistence of TMV in soil may play an important role in virus transmission [3–5]. Crops planted in areas where persistence in soil sources is possible are exposed to TMV and are therefore at great risk for infection [1].

Currently, a variety of techniques have been established for the detection of TMV, including biological assay on *Nicotiana tabacum* Xanthi-NC by necrotic lesions [6], ELISA [7], and RT-PCR [8]. Generally, biological indexing on differential hosts is time consuming and takes 4–7 days to complete. However, serological diagnosis may not provide the sensitivity that is needed to detect low-level of contaminated soil samples. Detection and identification of TMV with RT-PCR would provide not only greater sensitivity, but also a quantitative assay when used with real-time technology. Moreover, RT-PCR amplified products could be used for nucleotide sequencing to further confirm the virus detected, as well as its genetic relationship with other assigned strains. However, while RT-PCR assay has usually been applied to plant samples rather than to soil, the persistence of TMV in soil has been documented by ELISA and bioassay [9].

In this study, three different methods were assessed for their capacities for detecting TMV from soil. Additionally, the method of immunocapture real-time quantitative RT-PCR from soil for TMV detection was established, and immunocapture sample preparation was incorporated to permit the entire test, from sample preparation to amplicon detection, to be completed in a single tube.

## 2. Materials and Methods

### 2.1. Soil, Virus Strains and Plant Materials

The assays were developed and assessed with 68 soil samples collected from nine Chinese provinces where tobacco and tomato that were infected by TMV was planted. TMV was characterized and maintained in *Nicotina tabacum cv*. K326 in the authors’ laboratory. TMV particles were purified according to the method of Gooding and Herbert [10]. *Tomato mosaic virus* (ToMV) provided by professor Jiang Guoyong of Qingdao Agriculture University was used as negative control.

### 2.2. Artificial Contamination of Soil with TMV, and Extraction of RNA from Soil

To confirm the sensitivity of ELISA, RT-PCR and IC-real-time RT-PCR for detecting TMV in plant and soil, one gram aliquots of soil were treated with a series of TMV infected leaf sap dilutions of 1:10^1^, 1:10^2^, 1:10^3^, 1:10^4^, 1:10^5^ and 1:10^6^ (w/v, g/mL), respectively. One gram of air-dried TMV-contaminated or uncontaminated soil samples were suspended in 1 mL extraction buffer (0.01 M phosphate buffer pH 7.0) in Eppendorf tubes. The suspensions were agitated on a vortex mixer for 1 min, shaken on a rotary shaker (200 rpm) for 30 min at room temperature, incubated at 4 °C overnight, and then centrifuged at 10,000×g for 2 min. The supernatants were used for DAS-ELISA and IC-real-time RT-PCR. Based on Horm’s study [11], direct RNA extraction from 1 g of TMV-contaminated or uncontaminated soil and ToMV-contaminated soil were prepared using the QIAamp Viral RNA Mini Kit (Qiagen, Valencia, CA, USA) according to the manufacturer’s instruction. The same TMV infected leaf sap dilutions were directly investigated by DAS-ELISA, IC-real-time RT-PCR and total RNA extraction, respectively. Healthy leaf extract was used as negative control.

### 2.3. Enzyme-Linked Immunosorbent Assay (ELISA)

TMV antibody and its enzyme conjugate were supplied by ADGEN (ADGEN Diagnostic Systems, Ayr, Scotland, UK). Double antibody sandwich ELISA was carried out following the instruction of the manufacture. Leaf tissue or soil mentioned in Section 2.2 were prepared. Absorbance at 405 nm was measured with an ELISA reader, SpectraMax Plus 384 (Molecular Devices, Sunnyvale, CA, USA). A sample was considered positive if its absorbance reading was at least two times higher than that of the non-treated control sample (plant and soil).

### 2.4. The Conventional RT-PCR

First strand cDNA were synthesized by reverse transcribing total RNA of leaf tissue or soil using an Exscript^™^ RT reagent kit (TaKaRa, Dalian, China) according to the manufacturer’s instructions. The forward primer (5′-ATTAGACCCGCTAGTCACAGCAC-3′) and the reverse primer (5′-GTGGGGT TCGCCTGATTTT-3′) were designed using the *PerlPrimer* software based on the conserved nucleotide sequences of TMV *CP* in GenBank [12], which were determined based on the alignment of TMV *CP* RNA sequences using the DNASTAR package (Version 7.0, DNAStar Inc. Madison, WI, USA). The conventional PCR was performed using the above synthesized cDNA. The amplication was carried out in a 25 μL total reaction volume by using rTaq polymerase (TaKaRa) with 25 pmol of forward and reverse primers and 0.5 μL cDNA according to the manufacturer’s protocol. The thermal profile of PCR was the same as used for real-time PCR (see below), performed in a T3 Thermocycler (Biometra, Göttingen, Germany). The primers 5′-CAAGGAAATCACCGCTTTGG-3′ (forward) and 5′-AAGGGATGCGAGGATGGA-3′ (reverse) for the *Actin* gene of tobacco were used as internal controls.

### 2.5. Immunocapture Real-Time RT-PCR (IC-Real-Time RT-PCR)

Immunocapture was conducted following the method described by Jacobi [13]. Briefly, 0.2 mL thin-wall polypropylene PCR tubes in strips were coated with 30 μL of 1.0 μg/mL anti-TMV antibody (ADGEN Diagnostic Systems) in ELISA coating buffer and incubated for 2–4 h at 37 °C or overnight at 4 °C. The supernatant of soil suspension or plant sap was loaded in the anti-TMV antibody coated PCR tubes and inoculated for 2 h at 37 °C to allow TMV particles to be trapped to the tubes. After washing with phosphate washing buffer, the treated PCR tubes were ready for real-time RT-PCR. For each 25 μL reaction, 1 μL each of two sets of primers (in 10 μM stock) were added to 12.5 μL 2 × One Step SYBR RT-PCR Buffer, 1.5 μL TaKaRa Ex Taq HS Mix, 0.5 μL PrimeScript PLUS RTase Mix and 8.5 μL RNase free H_2_O. The thermal cycling process and fluorescence signal detection were carried out with the Applied Biosystems 7500 Real-Time PCR System (ABI, Foster City, CA, USA). The cycling parameters were: reverse transcription for 5 min at 42 °C, denaturation for 10 s at 95 °C, followed by 40 cycles of 10 s at 95 °C for denaturation and 36 s at 62 °C for annealing and extension. To generate a standard curve, three replicates of a series of 5-fold dilutions of purified virus (10 ng/mL, 2 ng/mL, 0.4 ng/mL, 0.08 ng/mL and 0.016 ng/mL) after immunocapture were amplified using One Step SYBR**^®^** PrimeScript**^®^** PLUS RT-PCR Kit. The standard curve was constructed using the cycle threshold values obtained against the known concentrations of serially diluted virus. The identities of the amplicons and the specificity of the reaction were verified by agarose gel electrophoresis and melting curve analysis, respectively. The relative RNA levels of TMV in different virus samples were computed with respect to the standard curve.

### 2.6. Statistical Analysis

Results were tested for significance using the Student’s *t*-test by parametric one-way analysis of variance (ANOVA), and those with *p*-values less than 0.05 were considered significant.

## 3. Results and Discussion

### 3.1. DAS-ELISA for TMV Detection in Soil and Plant

**Figure 1.**
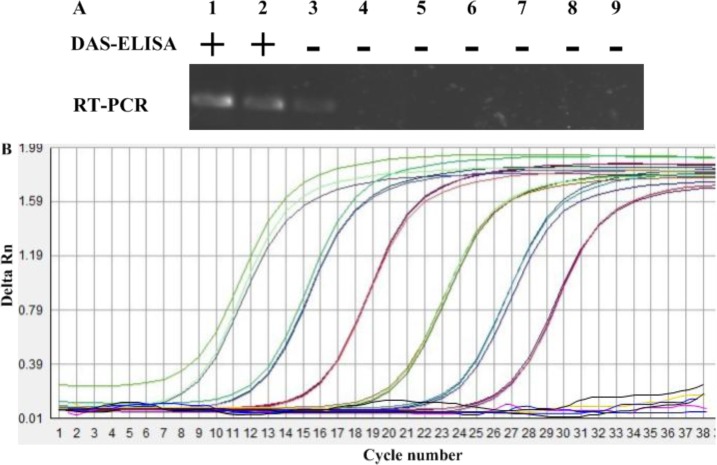
The detection of TMV in soil by the assays of DAS-ELISA (**A**), RT-PCR (**A**) and IC-real-time RT-PCR (**B**). Lanes 1 to 6 are the detections of TMV in one gram soil samples artificially blended with different dilutions naturally TMV-infected leaf at 1:10^1^, 1:10^2^, 1:10^3^, 1:10^4^, 1:10^5^ and 1:10^6^ (1 mL, w/v, g/mL), respectively, and, Lanes 7 to 9 are the detection of TMV from ToMV-contaminated soil, TMV-free soil, and healthy leaf extract, respectively. “+” represents positive result of DAS-ELISA for detecting TMV, “−” represents negative result of DAS-ELISA for detecting TMV. (B) Sensitivity of the IC-real-time RT-PCR assay as monitored by amplified curve. Shown from left to right are the curves of soil contamined with decreasing TMV-infected leaf saps at 1:10^1^, 1:10^2^, 1:10^3^, 1:10^4^, 1:10^5^ and 1:10^6^ (1 mL, w/v, per g soil) dilutions. The curves roughly parallel to the X axis are TMV from ToMV-contaminated soil, TMV-free soil, and healthy leaf extract, respectively.

**Figure 2.**
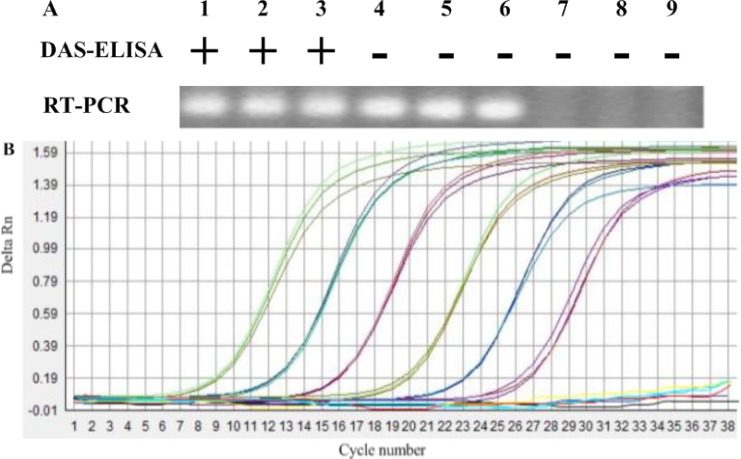
The detection of TMV in plant by the assays of DAS-ELISA (**A**), RT-PCR (**A**) and IC-real-time RT-PCR (**B**). Lanes 1 to 6 are dilutions at 1:10^1^, 1:10^2^, 1:10^3^, 1:10^4^, 1:10^5^ and 1:10^6^ (1 mL, w/v, per g soil) of naturally TMV-infected leaf saps, respectively, and, Lanes 7 to 9 are the template of ToMV-contaminated soil, TMV-free soil, and healthy leaf extract, respectively. “+” represents positive result of DAS-ELISA for detecting TMV, “−” represents negative result of DAS-ELISA for detecting TMV. (B) Sensitivity of the IC-real-time RT-PCR assay as monitored by amplified curve. Shown from left to right are the curves of decreasing TMV-infected leaf saps at 1:10^1^, 1:10^2^, 1:10^3^, 1:10^4^, 1:10^5^ and 1:10^6^ (1 mL, w/v, per g soil) dilutions. The curves roughly parallel to the X axis are TMV from ToMV-contaminated soil, TMV-free soil, and healthy leaf extract, respectively.

A serial of dilutions of 10^1^ to 10^6^ TMV-infected leaf sap (1 mL, w/v, g/mL) were added to one gram TMV-free soil, and those soils and above leaf saps were determined by DAS-ELISA, respectively. The results of three repeated tests indicated that the limit of DAS-ELISA to detect TMV in soil was diluted at 1:10^2^ (w/v, g/mL) (Figure 1). The results also indicated that the detection limit of TMV for TMV-infected leaf sap was diluted at 1:10^3^ (w/v, g/mL) (Figure 2). DAS-ELISA was more sensitive in plant materials than in soils.

### 3.2. Sensitivity of Conventional RT-PCR with Total Plant or Soil RNA Preparations

Using the optimized primer, sensitivities of conventional RT-PCR system for the detection of TMV were evaluated with two types of sample preparations, including infected plants and soil treated with TMV. The method showed the different detection limits for detection of TMV in plants or soil. With plants infected by TMV, a positive signal was detected down to the 1:10^6^ dilution. However, when a 10-fold dilution series of the same TMV-infected leaf sap were added to 1g air-dry soil, from which total RNA was extracted as described in Section 2.2, the sensitivity of conventional RT-PCR for detection in soil was only 1:10^3^ (Figure 1). The sensitivity of detection in plant extracts was thus about 10^3^ times higher than in soil (compare Figure 1 to Figure 2).

### 3.3. Sensitivity of IC-Real-Time RT-PCR to Detect TMV in Soil and Plant

Sensitivity of the IC-real-time RT-PCR to detect TMV in soil was evaluated using soil samples of one gram blended with dilutions of 1:10^1^, 1:10^2^, 1:10^3^, 1:10^4^, 1:10^5^ and 1:10^6^ of TMV-infected leaf (1 mL, w/v, g/mL). Tobacco leaf saps with the same dilution were also used to IC-real-time RT-PCR analysis. In this experiment, IC-real-time RT-PCR was capable of detecting TMV in all dilutions in three replicated tests of soil or plant (Figure 2). As the Figure 1 showed, positive bands were detected down to the 1:10^6^ dilution in TMV-contaminated soil, whereas, the dilution of 10^6^ in plants infected by TMV was detected by IC-real-time RT-PCR. Although sensitivity of the test may be even greater, we did not prepare dilutions beyond 1:10^6^. When the soil extracts at a dilution of 1:10^6^ TMV-infected sap were mechanically inoculated tobacco seedlings of NC89, no TMV was detected by RT-PCR at 7 dpi. Thus, a reliable detection of one gram TMV-infected leaf in 10^6^ gram soil was considered sufficient.

### 3.4. The Specificity of IC-Real-Time RT-PCR for the Detection of TMV in Soil

**Figure 3.**
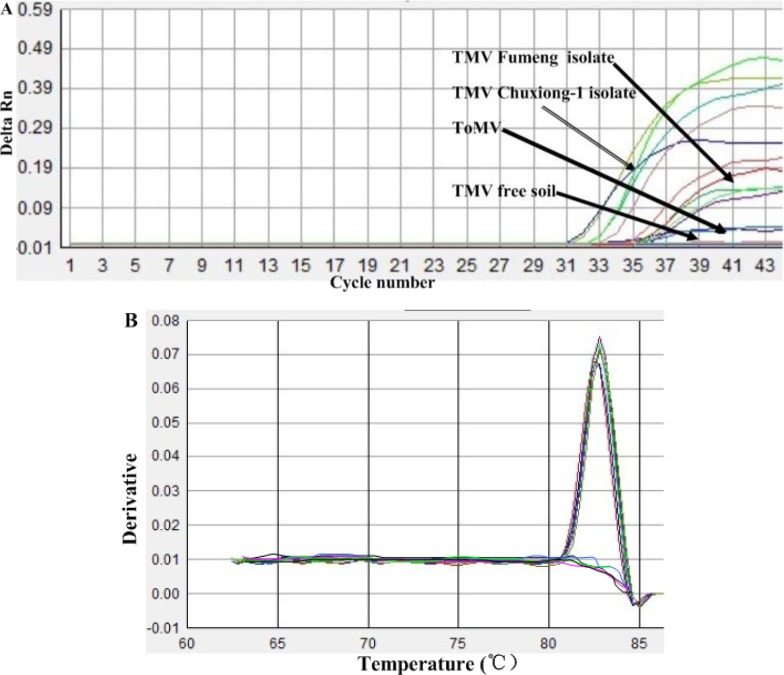
The specificity of IC-real-time RT-PCR for the detection of TMV in soil. The specificity of the IC-real-time RT-PCR assay as monitored by amplified curve (**A**) and melting curves (**B**) for TMV CP gene. (A) Amplified curve shown from left to right are the curves of TMV Fumeng isolate, TMV Chuxiong-1 isolate, ToMV and TMV-free soil. (B) Melting curves for TMV CP gene with single peak from three replicates of two different templates including TMV leaves and viruliferous soils.

**Figure 4.**
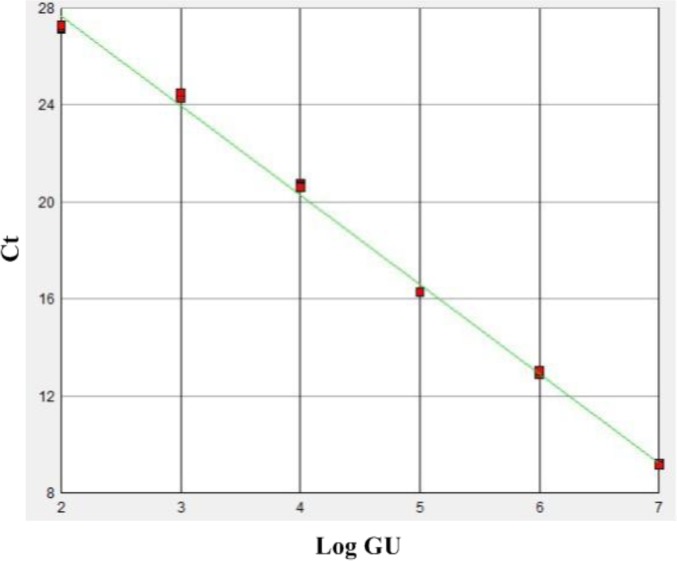
The standard curve obtained for the quantitation of purified TMV. The plot indicates the correlation between the Ct values and the log of the TMV particle units (Log GU). The equation of the regression line shown is y = −0.37x + 34.97; R^2^ = 0.99.

The specificity of the IC-real-time RT-PCR primers for the *CP* gene of TMV was established by ruling out cross-reactivity among virus-free soils and different virus species infecting identical hosts, such as ToMV. The TMV *CP* specific real-time RT-PCR primers, with a battery of reference isolates virus, including TMV Chuxiong-1 isolate (GenBank accession: HE818417) and TMV Fumeng isolate (GenBank accession: HE818416) isolate that represent two subpopulations of nature TMV populations in China, demonstrated a high degree of specificity for TMV by amplifying TMV only. The method yielded negative results on other viruses and virus-free soil tested (Figure 3(A)). In addition, the single wave crest of the melting curve from the IC-real-time RT-PCR assay also suggested that these primers had a high specificity to detect TMV in soil (Figure 3(B)). A liner relationship between the amount of input template with the concentrations of 1.0×10^3^, 1.0×10^2^, 1.0×10^1^, 1.0, 1.0×10^−1^, and 1.0×10^−2^ μg/mL TMV was obtained (Figure 4).

### 3.5. Comparison of ELISA, RT-PCR and IC-Real-Time RT-PCR for Detecting

Out of the 68 soil samples collected from nine provinces of China, in investigations performed after TMV disease outbreaks, seven samples (10.3%) tested positive by DAS-ELISA. Direct RNA extraction was performed on 68 samples with the QIAamp kit, and TMV viral RNA was detected in 16 samples (23.5%) by conventional RT-PCR. The IC-real-time RT-PCR was used to detect TMV from 68 samples without RNA extraction, and 27 samples (39.7%) tested positive (Table 1).

## 4. Discussion

Diagnosis of the pathogen plays an important role in crop improvement and disease management strategies. TMV was detected at 0–60 cm depths of clay textured soil 19 months after tobacco waste application [9]. TMV in soils serves as the most important sources for spread of TMV in subsequent crops. The aims of this work were to evaluate the capacities of three different methods including DAS-ELISA, conventional RT-PCR and IC-real-time RT-PCR to detect TMV in soil.

The ELISA method was used to detect TMV in clay soil [9]. Due to its simplicity and low-cost, antibody based ELISA testing is the most frequently used method in plant virus detection. However, ELISA assay need more time to complete. A further drawback of ELISA testing is the relatively low sensitivity compared to conventional RT-PCR; this is particularly pertinent for TMV testing in soils for tobacco planting where the virus level present is below the sensitivity of ELISA. This improved sensitivity has contributed to the proliferation of conventional RT-PCR assays in diagnostic laboratories. The sensitivity of the described conventional RT-PCR assay coupled with total RNA extraction was compared with ELISA and it was shown to be up to 10^3^- and 10^2^- times higher in plant materials and soils, respectively. When IC was used to prior to single-step real-time RT-PCR to detect TMV in plant saps and soils the sensitivity were 10^4^ and 10^3^ times higher relative to ELISA, respectively.

RT-PCR technology is widely used to detect viruses with RNA genomes, and has gained importance in recent years. Many new PCR methods have been developed, and have become routine for virus detection in the laboratory. Amplification in real-time PCR reactions is often affected by inhibitors present naturally in different matrices, the effect is often revealed by reduced amplification efficiency [14]. The efficiencies and good correlation coefficient for the real-time RT-PCR assays presented suggested that lack of detection of TMV from soil by RT-PCR may be due to inhibitors of PCR present in soil extract. Immunocapture of virions may well allow separation of the virus from potential inhibitors prior to dissociation of the virions to release RNA for the real-time PCR. Therefore, a limitation of single-step real-time RT-PCR assay is the inability to extract total RNA of TMV from soils contaminated by TMV. Direct extraction of total RNA from soil is a key procedure in RT-PCR and of great interest. In the past 20 years, many methods of RNA extraction from soil have been reported [15–20]. However, until now, there has been no method for RNA extraction from all type of soil, so researchers had to choose or develop soil RNA extraction methods to fit their own research purposes. The lack of a universal RNA extraction method for all soil soils hindered the ability to quantify the content of TMV in soil. Immunocapture RT-PCR (IC-RT-PCR) technique for virus detection, is a combination of immunology and RT-PCR technique developed by Jansen [21], an application of the specific interaction of antibody with antigen. The principle of this method is that first the specific antibody captures the viral antigen (in the form of virions), after which the RT-PCR reaction is initiated by heating to dissociate virions to release the genomic RNA without the necessity for total RNA extraction; thus the specificity and sensitivity of this method is very high. In the present study, the limit of IC-real-time RT-PCR was 10^2^-times higher than that of RT-PCR to detect TMV in soil, however, the sensitivity of both methods were similar to detect TMV in plant materials. TMV and ToMV are closely related, and both infect pepper, potato, tomato and several other hosts. Applying the IC-real-time RT-PCR could detect TMV in soil infected by TMV [22]. No positive result was found in virus-free or ToMV infected soil. This suggested that these antibody and primers had a high specificity in detecting TMV in soil.

## 5. Conclusions

In conclusion, a IC-real-time RT-PCR assay was used to detect TMV in soil. It was more rapid, sensitive, and specific than either the RT-PCR or DAS-ELISA assays. This assay method could help accurately determine the TMV content of soil. Based on the accurate determination of virus occurrence in soil, crop rotations could be utilized or resistant varieties planted, in order to control TMV and curtail viral disease outbreaks in the crop.

## Figures and Tables

**Table 1.** Detection of TMV in soil samples by DAS-ELISA, RT-PCR and IC-qRT-PCR.

	**DAS-ELISA**	**RT-PCR**	**IC-qRT-PCR**
TMV-positive number	7c	16b	27a
TMV-positive rate (%)	10.3c	23.5b	39.7a

Different letters indicate a significant difference of the means at *p* = 0.05 using the Student’s *t*-test by parametric one-way analysis of variance (ANOVA).
